# A Review of Pinealectomy-Induced Melatonin-Deficient Animal Models for the Study of Etiopathogenesis of Adolescent Idiopathic Scoliosis

**DOI:** 10.3390/ijms150916484

**Published:** 2014-09-18

**Authors:** Man Gene Chi Wai, Wang William Wei Jun, Yim Annie Po Yee, Wong Jack Ho, Ng Tzi Bun, Lam Tsz Ping, Lee Simon Kwong Man, Ng Bobby Kin Wah, Wang Chi Chiu, Qiu Yong, Cheng Jack Chun Yiu

**Affiliations:** 1 Department of Obstetrics and Gynaecology, Faculty of Medicine, the Chinese University of Hong Kong, Hong Kong, China; E-Mails: geneman@cuhk.edu.hk (M.G.C.W.); ccwang@cuhk.edu.hk (W.C.C.); 2 Department of Spine Surgery, Drum Tower Hospital, Nanjing University Medical School, Nanjing 210008, China; E-Mails: drwilliamwang@163.com (W.W.W.J.); scoliosis2002@sina.com (Q.Y.); 3 Joint Scoliosis Research Center of the Chinese University of Hong Kong and Nanjing University, Hong Kong, China; 4 Department of Orthopaedics & Traumatology, Faculty of Medicine, the Chinese University of Hong Kong, Hong Kong, China; E-Mails: anniepym@gmail.com (Y.A.P.Y.); tplam@ort.cuhk.edu.hk (L.T.P.); bobng@ort.cuhk.edu.hk (N.B.K.W.); 5 School of Biomedical Sciences, Faculty of Medicine, the Chinese University of Hong Kong, Hong Kong, China; E-Mails: jack1993@yahoo.com (W.J.H.); tzibunng@cuhk.edu.hk (N.T.B.); 6 Lee Hysan Clinical Research Laboratory, Faculty of Medicine, the Chinese University of Hong Kong, Hong Kong, China; E-Mail: simonlee@cuhk.edu.hk

**Keywords:** adolescent idiopathic scoliosis, melatonin, pinealectomy

## Abstract

Adolescent idiopathic scoliosis (AIS) is a common orthopedic disorder of unknown etiology and pathogenesis. Melatonin and melatonin pathway dysfunction has been widely suspected to play an important role in the pathogenesis. Many different types of animal models have been developed to induce experimental scoliosis mimicking the pathoanatomical features of idiopathic scoliosis in human. The scoliosis deformity was believed to be induced by pinealectomy and mediated through the resulting melatonin-deficiency. However, the lack of upright mechanical spinal loading and inherent rotational instability of the curvature render the similarity of these models to the human counterparts questionable. Different concerns have been raised challenging the scientific validity and limitations of each model. The objectives of this review follow the logical need to re-examine and compare the relevance and appropriateness of each of the animal models that have been used for studying the etiopathogenesis of adolescent idiopathic scoliosis in human in the past 15 to 20 years.

## 1. Introduction

Adolescent idiopathic scoliosis (AIS) is a complex three-dimensional structural deformity of the spine, characterized by vertebral rotation in the transverse plane, lateral curvature in the frontal plane and, very often, also, abnormal alignment in the sagittal plane. This phenomenon is only known to occur in human, with a higher susceptibility in the female population. The prevalence of AIS ranges from 2% to 4% worldwide [1,2,3], and about 10% of AIS patients have significant deformity that would require treatment [3,4]. The spine deformity has been reported to cause a disturbed self-image and potential health problems associated with cardiopulmonary function and back pain problem in AIS girls exhibiting severe progressive curves. The current bracing and surgical treatments have significant limitations and associated morbidities. Proper elucidation of the etiology and pathogenetic mechanisms of AIS is essential for effective prediction of the occurrence, prognosticating, prevention and treatment. To further understand the etiopathogenesis of AIS, different types of scoliosis-induced experimental animal models have been developed, and have since been used for more than a century [5,6,7]. Each of the animal models found in the literature specifically focuses on one of the many different possible hypotheses on the pathogenesis of AIS. However, with the many different hypotheses having been proposed in recent years, melatonin deficiency [8,9] and melatonin signaling pathway dysfunction [10,11] have received significant attention and proposed as possible contributory factors in the etiopathogenesis of AIS. In order to prove this hypothesis, different animal models have been developed in order to unravel this enigma. Such animal models include the model with surgical removal of the pineal gland [12,13,14,15] and the naturally melatonin deficient mouse model [16].

**Figure 1:**
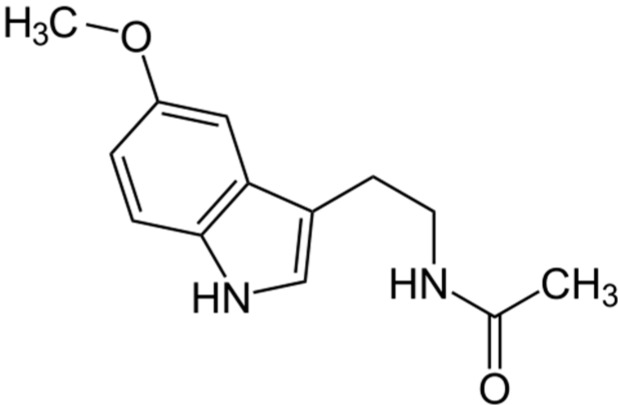
Structure of melatonin.

Melatonin, also known as *N*-acetyl-5-methoxytryptamine, is the major hormone secreted by the pineal gland (Figure 1) [17]. In order to study the role of melatonin in the etiopathogenesis of idiopathic scoliosis, a sizeable volume of literature has described the use of various experimentally induced melatonin-deficient animal models of scoliosis. The first experimental scoliosis model related to melatonin was established in 1959 by Thillard. In this model, post-operative spinal curvature was observed in chickens following surgical removal of the pineal gland [18]. Following this report, numerous experimental procedures employing different animals have been used. However, the variety of procedures and models used make it extremely difficult to determine the appropriateness of each animal model on their relevance in clarifying the role of melatonin in the etiopathogenesis of idiopathic scoliosis in human.

The purpose of this study was to provide an overview of melatonin-deficient experimental animal models used in studying the etiopathogenesis of AIS. In addition, the usefulness and limitations of each model and the relevance of the respective findings, in contributing to the further understanding of AIS in human will be discussed.

## 2. Pinealectomized Avian (Chicken) Model

**Figure 2:**
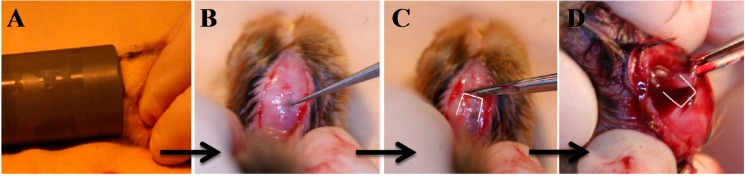
Pinealectomy performed on a three-day-old chick. (**A**) Under isoflurane inhalation, chicken at three days post-hatching was subjected to general anesthesia for pinealectomy (PINX); and (**B**–**D**) The back of the cranium was cut open and the pineal gland was removed.

The term pinealectomy (PINX) refers to the surgical ablation of the pineal gland (Figure 2). Thillard was credited with the development of the first PINX model in 1959 [18]. By removing the pineal gland, post-operative spinal curvature was observed in 65% of the operated chickens. It was not until 1983 that Machida and Duboussett popularized this model by drawing a morphological correlation with AIS in patients [12]. In their study, young chicks developed a three-dimensional spinal deformity consisting of lateral curvature with vertebral body rotation two weeks after PINX. Ninety chickens were divided into three groups: (1) chickens that had undergone PINX; (2) PINX chickens with an autografted pineal gland in the intramuscular tissue of the trunk; and (3) chickens receiving no treatment serving as the control. At the end of two weeks, scoliosis was reported in 100%, 10%, and 0% of the chickens in each group, respectively. This led to the hypothesis that neurotransmitters or neurohormonal systems in the pineal body constituted a major factor in this type of experimental scoliosis.

**Figure 3:**
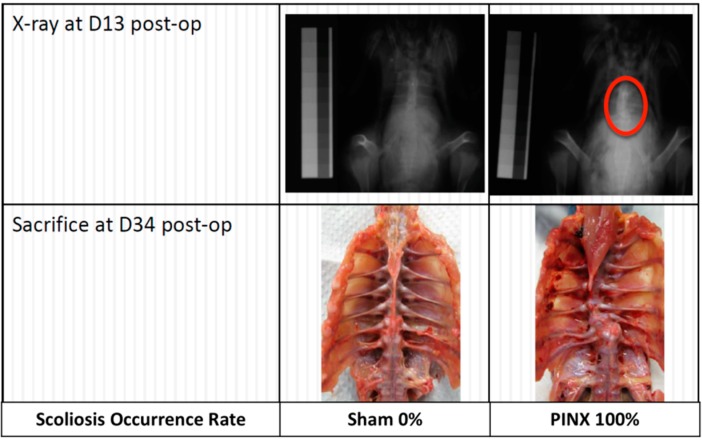
Occurrence of scoliosis in Kamei chickens. Unisex local bred Kamei chickens were pinealectomized (PINX) three days after hatching. X-ray taken thirteen days after the operation revealed a development of spinal curvature (encircled in red). At 34 days post-operatively, the chickens were sacrificed and autopsy was conducted to evaluate the incidence of scoliosis. All of the chickens in the PINX group demonstrated scoliosis (*n* = 7), while no scoliosis was detected in the sham-operated control group (*n* = 6).

The observation that the main secretory product of the pineal gland is melatonin has helped to bring forward a new hypothesis that melatonin deficiency is associated with spinal deformity. To prove this, Machida *et al.* studied the effect of melatonin and serotonin supplementation in PINX chickens [19]. A total of 90 White Leghorn chickens underwent PINX on the third day after hatching, and were allocated into three groups of the same size: (1) group treated with serotonin, daily for 21 days; (2) group treated with melatonin, daily for 21 days; and (3) control group without any treatment. The results showed that scoliosis developed in 100% of chickens in the control group, 73% of those treated with serotonin and 20% of those treated with melatonin. In addition, the six melatonin-treated chickens with scoliosis showed less severe curvatures than the other two groups. They concluded that melatonin metabolism played an essential role in the development of scoliosis. They subsequently reported that the overall incidence of scoliosis varied from 80% to 100% in PINX chickens [12,19,20,21,22,23,24]. In a similar study, our group also observed a similar percentage of scoliosis in chickens when PINX was performed three days after hatching (Figure 3) (unpublished data). However, the occurrence of scoliosis reported by other researchers varied considerably (Table 1) [13,15,25,26,27,28,29,30,31,32,33,34,35,36,37,38,39,40,41,42]. The most notable study was conducted by Bagnall *et al.*, in 1999 [25], in which 105 newly hatched Mountain Hubbard chickens were used. Three days after hatching, 89 chickens underwent PINX, and were subsequently divided into four groups for treatment: (1) 23 PINX chickens receiving melatonin (2.5 mg/kg per body weight (BW)); (2) 23 PINX chickens receiving vehicle (control); (3) 22 chickens receiving melatonin (2.5 mg/kg BW) after 14 days of PINX post-operatively; and (4) 22 PINX chickens receiving no treatment at all. The remaining 16 chickens, which did not receive PINX or other treatments, served as normal controls. After five weeks of post-PINX melatonin treatment, over 50% of the chickens still developed scoliosis leading to the observation that melatonin therapy might not effectively lower the incidence or severity of scoliosis in the PINX chickens. Likewise, the incidence of scoliosis in the PINX chickens (57%) was much lower than what had been reported by Machida *et al.* [19]. Although the dosage of melatonin used was substantially lower but more physiological than that used by Machida *et al.* (2.5 mg/100 mg BW) [19], it was argued that the melatonin dosage administered might be insufficient and had not been adjusted to take into account the rapid weight gain of the growing chickens [25]. A number of other studies also contradicted the finding on the positive effect of intramuscularly autografted pineal body in preventing the occurrence of scoliosis (Table 2) [26,32]. It was also doubtful whether the melatonin level could be sustained by the auto-transplanted pineal body and had an adequate effect on curve resolution.

**Table 1:** Different varieties of melatonin-deficient animal models.

Procedure	Animal	Species/Variety	% of Animals with Scoliosis	Age at Operation	Time after Procedure at Which Scoliosis Was Diagnosed	Reference
Pinealectomy (PINX)	Chicken	Leghorn White	100% (30/30)	/	14 days	[12]
		Leghorn White	82% (36/44) in male, 100% (6/6) in female	2 days	90 days	[21]
		Leghorn White	100% (30/30)	3 days	14 days	[19]
		Leghorn White	48% (10/21)	3 days	14 days	[31]
		/	85% (17/20)	3 days	14 days	[13]
		Leghorn White (Male)	100% (40/40)	2 days	14 days	[23]
		Leghorn White	50% (15/30)	3 days	21 days	[33]
		Leghorn White	52% (17/33)	3 days	14 days	[34]
		Mountain Hubbard	57% (12/21)	3 days	21 days	[25]
		Leghorn White	26% (9/35)	3 days	14 days (increased to 60% (21/35) after 35 days)	[28]
		Leghorn White	25% (5/20)	3–5 days	14 days (increased to 55% (11/20) after 35 days)	[27]
		Leghorn White (Female)	45.5% (10/22)	2 days	28 days (increased to 63.6% (14/22) after 84 days)	[29]
			45.5% (10/22)	4 days	28 days (increased to 72.7% (16/22) after 84 days)	
			38.1% (8/21)	11 days	28 days (increased to 81% (17/21) after 84 days)	
			10% (2/20)	18 days	28 days (increased to 70% (14/20) after 84 days)	
		Leghorn White	95% (19/20)	2 days	14 days	[22]
		/	58% (21/36)	7 days	35 days	[26]
		Mountain Hubbard	50% (10/20)	/	22 days	[30]
		Leghorn White	53.8% (7/13)	3 days	42 days	[35]
		Leghorn White	25% (13/25)	3 days	90 days	[36]
		Hybro Broiler	58% (7/12)	2 days	56 days	[32]
		Leghorn White	100% (15/15)	2 days	90 days	[24]
		Hybro Broiler (Female)	93.6% (87/93)	3 days	14 days	[42]
			90% (9/10)		6 days	
		Leghorn White	50% (11/22)	3 days	42 days	[37]
		Hybro Broiler	93% (14/15)	3 days	56 days	[15]
		Leghorn White	42% (25/59)	2 days	35 days (increased to 45% (24/53) after 70 days	[40]
		/(Female)	95% (20/21)	3 days	35 days	[39]
		Steggles	75% (30/40)	2 days	14 days	[38]
		Hybro Broiler (Female)	84.2% (16/19)	3 days	7 days	[41]
			88.9% (16/18)		14 days	
			89.5% (17/19)		21 days	
		Hybro Broiler (Female)	100% (10/10)	3 days	60 days	[20]
	Rat	Sprague-Dawley	0% (0/32)	2–4 days	44 days	[31]
		Sprague-Dawley (Male)	0% (0/10)	21 days	90 days	[14]
	Hamster	Syrian	0% (0/17)	11–13 days	43 days	[31]
	Salmon	Atlantic	82% (71/86)	3 years	42 days	[43]
	Monkey	Rhesus	0% (0/18)	8–11 months	300–1230 days	[44]
Intense Continuous Lighting	Chicken	Mountain Hubbard	15% (3/20)	/	22 days	[30]
		Leghorn White	0% (0/41)	3 days	77 days	[35]
Intense Continuous Lighting + PINX	Chicken	Mountain Hubbard	80% (16/20)	/	22 days	[30]
Bipedalism + PINX	Rat	Sprague-Dawley (Male)	100% (20/20)	21 days	90 days	[45]
			100% (10/10)	21 days	90 days	[14]
	Mouse	C3H/HeJ (Male)	70%	35 days for bipedalism; 42 days for PINX	315 days	[46]
Bipedalism	Rat	Sprague-Dawley (Male)	0 (0/5)	21 days	90 days	[45]
			0 (0/10)	21 days	90 days	[14]
	Mouse	C57BL/6J	97% (29/30)	21 days	150 days	[16]
		C57BL/6J (Male)	64.3%	35 days	315 days	[46]
		C3H/HeJ (Male)	25%	35 days	315 days	[46]
Quadrupedal + PINX	Rat	Sprague-Dawley (Male)	0 (0/10)	21 days	90 days	[45]
Natural (Congenital) Model	Mouse	C57BL/6J	25% (5/20)	/	150 days	[16]

## 3. Pinealectomized Bipedal Rodent (Rat) Model

To evaluate whether a phenomenon similar to those observed in chickens could be observed in mammals, O’Kelly *et al.* performed PINX in quadrupedal rodents (rats and hamsters) to evaluate the occurrence of scoliosis [31]. However, in contrast to the avian models, PINX alone was not able to induce spinal curvature in any of these rodent models, [12,19,21,23]. This led to the theory that the quadrupedal rodent spine unlike the bipedal chickens and human is not subjected to the same postural and dynamic mechanical forces essential for the development of scoliosis. Hence, this led to the introduction of the bipedal rat model. The bipedal rat model was created by amputating both forearms and the tail at three weeks of age. Subsequently, the upright posture was further stimulated by gradually raising the food and water provided to a higher level. To confirm this, Machida *et al.* further created several rat models: (1) sham-operated bipedal; (2) pinealectomized quadrupedal; (3) pinealectomized bipedal; and (4) pinealectomized with implanted melatonin pellets, to prove the speculation [45]. It was discovered that only the pinealectomized bipedal rats developed scoliosis at around three months post-operatively. Moreover, treatment with a melatonin pellet (100 mg per 90 days release) could prevent the development of scoliosis in nine out of 10 pinealectomized bipedal rats. It was concluded that any disturbance on the equilibrium and other postural mechanisms secondary to a deficiency of melatonin after pinealectomy may promote the development of lordoscoliosis with vertebral rotation, especially in the bipedal posture, which might then progress.

## 4. Congenital Melatonin-Deficient Rodent (Mouse) Model

To further demonstrate the relationship between melatonin and induction of scoliosis, an *AA-NAT* gene knock-out strain of mice with congenital melatonin deficiency (C57BL/6J) was developed. [47]. The mice lack the *AA-NAT* gene, a key enzyme for the biosynthesis of melatonin from serotonin, and exhibit a depressed melatonin level in plasma and pineal gland [47]. In addition, many studies have found the lowest bone mineral density (BMD) in C57BL/6J mice when compared with the various inbred mouse species and, thus, could be an ideal model for assessing the effect of melatonin on bone formation *in vivo* [48,49,50,51,52].

Machida *et al.* utilized C57BL/6J mice with surgically-induced bipedalism to assess the rate of development of scoliosis and the effect of daily injections of melatonin (8 mg/kg) in reversing scoliosis development [16]. After five months of treatment, the mice were sacrificed and the spine was examined by X-ray and 3D computed tomography (CT). The results indicated that nearly 100% of the melatonin-deficient bipedal mice developed spinal deformity, compared with 25% of the quadrupedal mouse model. They also reported that the treatment with exogenous melatonin was able to prevent the development of scoliosis in both models.

In line with the previous studies, the same team extended their study [46] by removing the forelimbs of these mice, without performing PINX, and achieved the induction of scoliosis at a higher rate (Table 1). The relationship between melatonin and scoliosis development was further illustrated by utilizing melatonin-proficient C3H/HeJ mice. In brief, when the forelimbs of the mice were amputated, the occurrence of scoliosis was two-fold lower compared with the bipedal C57BL/6J mice. But when PINX was performed in C3H/HeJ mice, the incidence of scoliosis increased to 70%. The results appeared to be in concordance with earlier reports that spinal deformity occurred under the conditions of melatonin deficiency and bipedal amputation.

**Table 2:** Effect of exogenous melatonin on inhibiting scoliosis development in different animals.

Procedure	Animal	Strain/Variety	Treatment (Melatonin/Melatonin Precursor/Pineal Transplantation)	Dosage of Melatonin	Duration of Melatonin Treatment	% of Melatonin-Treated Animals with Scoliosis	% of Animals Without Melatonin Treatment Demonstrating Scoliosis	Reference
Pinealectomy (PINX)	Chicken	Leghorn White	Serotonin	1.5 mg/100 mg/every other day; *i.p.*	21 days	73% (22/30)	100% (30/30)	[19]
			Melatonin	2.5 mg/100 mg/every other day; *i.p.*		20% (6/30)		
		Leghorn White (Male)	5-hydroxytryptophan	100 mg/100 mg/twice daily; *i.p.*	84 days	70% (28/40)	100% (40/40)	[23]
		Mountain Hubbard	Melatonin	2.5 mg/100 mg/daily; *i.p.*	35 days after PINX	52% (12/23)	57% (12/21)	[25]
				2.5 mg/100 mg/daily; *i.p.*	21 days (started after PINX for 14 days)	55% (12/22)		
		Hybro Broiler (Female)	Melatonin	8 mg/kg BW/daily; *s.c.*	56 days	20% (2/10)	100% (10/10)	[20]
		Leghorn White	Transplantation of pineal gland; *i.m.*	/	14 days	10% (3/30)	100% (30/30)	[12]
		/	Transplantation of pineal gland; *i.m.*	/	35 days	46% (17/37)	58% (21/36)	[26]
		Hybro Broiler	Transplantation of pineal gland; *i.m.*	/	56 days	50% (6/12)	58% (7/12)	[32]
Bipedalism	Rat	Sprague-Dawley (Male)	Melatonin pellet	100/90 days release	90 days	10% (1/10)	90% (9/10)	[45]
	Mice	C57BL/6J	Melatonin	8 mg/kg BW/daily; *s.c.*	140 days	0 (0/30)	97% (29/30)	[16]
Natural Model	Mice	C57BL/6J	Melatonin	8 mg/kg BW/daily; *s.c.*	140 days	0 (0/20)	25% (5/20)	[16]

## 5. Pinealectomized Fish (Salmon) Model

The previous findings indicated that posture itself may be one of the key factors in the development of scoliosis. Hence, the question remains on how melatonin deficiency alone could promote the occurrence of scoliosis. To resolve this, a study was conducted to evaluate the long-term effects of surgical ablation of the pineal gland on the spine of Atlantic salmon [43]. Unlike the avian and rodent models, the salmon spine is not weight bearing due to the buoyancy provided by the swim bladder and the density of the water thus eliminating the effect of bipedal posture. In addition, it would be easier to observe any morphological changes in the salmon spine which is straight and without marked regional specializations. The spinal movement of salmon is mainly limited to lateral flexion. Moreover, with the acellular bone tissue of teleosts, the loss of mineralization of the spine can be evaluated on the surface of the bone. In this study, the results showed 82% of the pinealectomized fish developed abnormal spinal curvatures. Evaluation of the individual vertebral bodies revealed significantly lower mechanical properties indexes in stiffness, yield limit and resilience than those in the sham controls. Moreover, the calcium, phosphorus, and total mineral content of the vertebral bodies were also significantly lower in the pinealectomized fish. The observed alterations of the spinal curve accompanied by changes in the proportions, mechanical strength and mineral content of the vertebral bodies following removal of the pineal gland in the salmon reinforced the hypothesis that melatonin could play a pivotal role in vertebral bone growth, bone mineralization and development of scoliosis.

## 6. Pinealectomized Non-Human Primate (Monkey) Model

Prompted by the success of these experiments, interest in non-human primates began to develop. In 2005, Cheung *et al.* performed PINX in bipedal non-human primates [44]. Among the 18 pinealectomized rhesus monkeys, 10 exhibited a significant loss of melatonin secretion, yet none of them developed the anticipated scoliosis in the post-operative follow-up period of 29 months. The study was criticized however, on the ground that the natural posture of the monkeys was restricted, as they were not allowed to move freely or stand upright because of space limitation in the cages [53]. Thus, similar to the situation in the quadrupedal rats, there was no gravitational pressure on the spine to induce the development of scoliosis. These findings further emphasize in the posture and posturally related mechanical forces and not melatonin deficiency alone, could be crucial in the development of scoliosis.

## 7. Discussion

Review of the literature showed consistently that scoliosis-like deformity can be induced from different types of melatonin-deficient animal models. However, many controversial issues remain in regards to each of the models. The incidence of scoliosis in chickens after PINX varied greatly among different studies (Table 1). The concern remained whether the deprivation of melatonin or the pinealectomy operation itself, could be the primary factor contributing to the development of induced scoliosis. To further evaluate this, studies on different resection techniques and the possible associated damage to the adjacent neural structures were conducted [27,28]. The findings revealed that cutting the pineal stalk could be the crucial step in the induction of scoliosis by PINX, rather than the removal of the gland or other artifacts arising from the surgery. Another study succeeded in demonstrating an increased occurrence of scoliosis in normal chickens exposed to continuous intense lighting [30]. Melatonin production was suppressed by continuous intense lighting and scoliosis was induced in 15% of the chickens. The employment of the combination of PINX and continuous intense lighting further increased the occurrence of scoliosis from 50% to 80% [30]. This led to the hypothesis that a certain threshold of melatonin deficiency might be needed to initiate the development of scoliosis. However, this observation could not be reproduced by Cheung *et al.* [35].

Another important concern is whether the age after hatching at which PINX is performed will affect the incidence of PINX-induced scoliosis in the chicken. Illés and Horváth have reported scoliosis occurrence in 80% of the chicken with PINX on the first day of hatching [54]. The number dropped to 70%, 50%, 30% and 0% when the same procedure was performed on the second, third, fourth and fifth day after hatching respectively. This was also confirmed by Inoh *et al.* who found 45.5% of the chicken had developed scoliosis after 1 month if PINX was performed at 4 days after hatching, compared to only 38.1% and 10% with surgery done at 11 and 18 days after hatching, respectively [29]. Although the occurrence of scoliosis in each of the different groups was similar at 12 weeks post-operation, the severity was more prominent in those with PINX performed at an earlier age. However, Bagnall *et al.* did not find statistically significant variation in the incidence of scoliosis with the age at PINX in the chicken though more chickens actually developed scoliosis 2 weeks after PINX when the surgery was performed earlier than 3 days after hatching [54].

The third main concern is about the appropriateness of the chicken and salmon model for studying scoliosis as there are fundamental differences in the anatomy and biomechanical loading of the avian/fish spine from human spine. The chicken spine shows only eight thoracic vertebrae with fused lumbar vertebrae and only two intervertebral spaces with marked intervertebral discs that can be regarded as mobile segments [42]. The thoracic and lumbar spine is positioned horizontally within the trunk and biomechanically distinct from the erect human spine. Most importantly, the dissimilarity between avian and mammalian bone architectures (e.g., lower bone mineral density and hollow trabecular center in avian bone) remains the most serious concern. For the Atlantic salmon model, there was an even a greater concern when its spine is compared with the human spine. Firstly, in this species, it is only phylogenetically distantly related to avians and mammals; Secondly, the spine is not weight bearing due to the buoyancy provided by the swim bladder and the density of the water; Thirdly, there is no marked regional specialization of the spine, and movement is only limited to lateral flexion. These traits already generate a skeptical view on how it can truly be compared with the specialized, segmented and gravitationally loaded human spine.

Despite the success in inducing scoliosis in chickens, the mechanism of how melatonin deficiency leads to the development of scoliosis remained uncertain. Studies on other more phylogenetically related animals (e.g., rats and hamsters) failed to produce scoliosis after PINX. Non-human primates are phylogenetically closest to man and, thus, should be the most ideal model for investigation [31]. However, there are still differences in this model compared with human postural movements. Non-human primates move with a semi-erect posture. Although their vertebral bodies closely resemble their human counterparts, their spine is not biomechanically loaded in a manner similar to the human spine [55]. The study using primates failed to produce the scoliosis observed in the avian and mouse models [44]. Researchers argued that this could be due to an inadequacy in the experimental design; the small cages have limited the movements and upright posture of the monkeys with the consequence that the much needed biomechanical stress for scoliosis development after PINX could not be induced [53]. However, it does cast doubt on whether the positive findings in less phylogenetically and biomechanically related animals can be extrapolated to the human. Even in the AIS patients, a low melatonin level has not been consistently documented [9,56,57]. Whether melatonin supplementation to AIS patients could affect the curvature progression also remains uncertain [9].

In addition, there is also a major concern on whether the anatomical development of the induced scoliosis in these animal models is different from that found in AIS patients. The study conducted by Machida *et al.*, demonstrated scoliosis with right convexity and dissymmetry of thoracic cage in bipedal melatonin-deficient mice after five months of surgery by 3D-computed tomography (CT) [16]. The occurrence of the convexity and dissymmetry would generate a similar three-dimensional deformity of the spine found in AIS patients [58]. Similarly, Machida *et al.* conducted another experiment to show the microarchitecture of low bone mineral density (BMD) in the scoliotic chicken models [20]. In brief, the results showed that post-mortem PINX chicken developed thoracic scoliosis and had a generalized lower cervical BMD than those without PINX by micro-CT. Most interestingly, the group with PINX and daily administration of exogenous melatonin prevented the loss of bone mineral density. The phenomenon on the generalized low BMD is very similar to those in AIS patient, as this has been commonly reported [59,60,61]. Based on other studies, there was a strong indication of the role of melatonin in bone formation, inducing proliferation and stimulation of type I collagen synthesis in primary human osteoblasts [62,63] and enhancing osteoid mineralization and osteoblast differentiation in mice and rat cell cultures [64]. The increase in bone mass was also observed after administration of melatonin *in vivo* [65,66,67]. In our studies, it was found that osteoblasts cultured from human AIS patients failed to respond normally to melatonin challenge at various doses with regard to proliferation and differentiation [68,69,70] in contrast to that of the normal controls. Despite this well-known clinical manifestation of AIS, its pathogenesis and relationship to premature osteoporosis remain unresolved. Although the properties of the bone microarchitecture in these scoliotic animal models are similar to the AIS patients, none of them exhibited multiple curvatures when melatonin is absent or deficient. It can be argued that the occurrence of multiple curvatures in human is due to the posture and mechanical stress imposed to seek balance. However, this would raise a question on the similarity of the bipedal animal model to the upright posture in human. Thus, it may indicate that the absence or deficiency of melatonin can only account for the manifestation of scoliosis, but not the possible mechanistic properties associated with the spinal curvature balance. Nevertheless, it would certainly be of great interest to elucidate whether the low BMD would occur in the melatonin-deficient rodents.

Evidently, there is no perfect animal model for the study of the etiopathogenesis of AIS in human at this stage. The animal models did however, trigger a substantial amount of research and advance our understanding of the role of melatonin and melatonin pathway in normal physiology and in particular skeletal growth, vertebral growth, bone metabolism and possible interaction with mechanical and other factors that affect spine development.

## 8. Conclusions

Though the precise mechanism(s) leading to the development of experimental scoliosis in the different animal models after PINX or melatonin deficiency remain(s) unknown, it is generally believed that melatonin has a significant contributory role. However, the exact function of melatonin or related melatonin pathway dysfunction in the etiopathogenesis of AIS in human still awaits many more in-depth studies.
