# Correction: Wai, M.G.C., *et al.* A Review of Pinealectomy-Induced Melatonin-Deficient Animal Models for the Study of Etiopathogenesis of Adolescent Idiopathic Scoliosis. *Int. J. Mol. Sci.* 2014, *15,* 16484–16499

**DOI:** 10.3390/ijms16023017

**Published:** 2015-01-29

**Authors:** Gene Chi Wai Man, William Wei Jun Wang, Annie Po Yee Yim, Jack Ho Wong, Tzi Bun Ng, Tsz Ping Lam, Simon Kwong Man Lee, Bobby Kin Wah Ng, Chi Chiu Wang, Yong Qiu, Jack Chun Yiu Cheng

**Affiliations:** 1 Department of Obstetrics and Gynaecology, Faculty of Medicine, The Chinese University of Hong Kong, Hong Kong, China; E-Mails: geneman@cuhk.edu.hk (G.C.W.M.); ccwang@cuhk.edu.hk (C.C.W.); 2 Department of Spine Surgery, Drum Tower Hospital, Nanjing University Medical School, Nanjing 210008, China; E-Mails: drwilliamwang@163.com (W.W.J.W.); scoliosis2002@sina.com (Y.Q.); 3 Joint Scoliosis Research Center of the Chinese University of Hong Kong and Nanjing University, Hong Kong, China; 4 Department of Orthopaedics and Traumatology, Faculty of Medicine, The Chinese University of Hong Kong, Hong Kong, China; E-Mails: anniepym@gmail.com (A.P.Y.Y.); tplam@ort.cuhk.edu.hk (T.P.L.); bobng@ort.cuhk.edu.hk (B.K.W.N.); 5 School of Biomedical Sciences, Faculty of Medicine, The Chinese University of Hong Kong, Hong Kong, China; E-Mails: jack1993@yahoo.com (J.H.W.); tzibunng@cuhk.edu.hk (T.B.N.); 6 Lee Hysan Clinical Research Laboratory, Faculty of Medicine, The Chinese University of Hong Kong, Hong Kong, China; E-Mail: simonlee@cuhk.edu.hk

The authors wish to make the following corrections to this paper [1]:

The first name and surname of the authors were reversed. It should be corrected in the following format (with the surname in bold text):

Gene Chi Wai **Man**, William Wei Jun **Wang**, Annie Po Yee **Yim**, Jack Ho **Wong**, Tzi Bun **Ng**, Tsz Ping **Lam**, Simon Kwong Man **Lee**, Bobby Kin Wah **Ng**, Chi Chiu **Wang**, Yong **Qiu** and Jack Chun Yiu **Cheng**

The authors would like to apologize for any inconvenience caused to the readers by these changes.
